# Coronary computed tomography angiography detection of short- and long-term outcomes after heart valve surgery with high risk cardiovascular patients

**DOI:** 10.1042/BSR20171450

**Published:** 2018-03-09

**Authors:** Zhi Zhu, Shuofeng Li

**Affiliations:** Department of Radiology, Cangzhou Central Hospital, Cangzhou 061001, Hebei Province, China

**Keywords:** Coronary computed tomography angiography (CCTA), Concomitant coronary artery disease (CAD), heart valve surgery

## Abstract

Coronary computed tomography angiography (CCTA) is a promising alternative technique to detect significant coronary artery lesions in high-risk cardiovascular patients with left ventricular dysfunction (left ventricular ejection fractions < 40%) referred for elective valve surgery, while little research about the use of CCTA to detect the outcomes of heart valve surgery was performed. Forty-six consecutive high-risk cardiovascular patients with the New York Heart Association (NYHA) classification were retrospectively studied. Immediate, 10-week, 20-week, and 40-week outcomes after heart valve surgery were assessed with CCTA. Patients’ average age at the time of surgery was 73 years, with the majority being male (54.35%). Among the CCTA parameters detected after 10, 20, and 40 weeks after heart valve surgery, only segment involvement score (SIS) did reach statistical significance when compared with baseline levels. The cumulative mortality rate at 10, 20, and 40 weeks were 19.56%, 30.43%, and 39.13% respectively. It can be seen that the early death is mainly due to complications, and with the time-lapse of surgery, the impact of complications on death is gradually eliminated. CCTA might be a useful tool to detect the outcomes of short- and long-term outcomes after heart valve surgery with high risk cardiovascular patients, and SIS level is associated with the short- and long-term outcomes.

## Introduction

Concomitant coronary artery disease (CAD), left ventricular dysfunction, and preoperative symptoms of advanced congestive heart failure portend an increased risk of worse clinical outcomes in patients undergoing heart valve surgery. Such high risk cardiovascular patient that requires surgery is not uncommon in industrialized countries [1] and various clinical studies have suggested that combined valve and bypass surgery could reduce early and late mortality [2–6]. According to the guidelines for the management of heart valvular disease, it was recommend that preoperative cardiac catheterization for the detection of coronary artery disease (CAD) should be done in male patients (age 35 or older), in postmenopausal women, and in premenopausal women with any risk factors for CAD [1,6].

Although it can be expensive and time consuming, preoperative cardiac catheterization remains the gold standard for the identification of CAD related coronary stenoses. However, such procedure may impart a small (0.1–0.2%) risk of catheter-related complications, such as death, myocardial infarction and stroke, thus a primary noninvasive technique as an alternative to preoperative cardiac catheterization in cardiovascular patients especially with high risk referred for valve surgery is therefore highly desirable.

Coronary computed tomography angiography (CCTA) has been shown to be a well-established imaging technique for detection atherosclerotic plaques and to evaluate the extent and the severity of coronary artery stenosis with high sensitivity and specificity [7] to assist in risk stratification [8,9]. Several studies have also demonstrated that CCTA can provide diagnostic capability and, most importantly, incremental prognostic value than calcium scoring for patients with suspected CAD [10]. Most studies in recent years have tested the diagnostic performance of CCTA in patients undergoing cardiac valvular surgical treatment [11], and there is little such research to detect the short- and long-term outcomes after heart valve surgery with high risk cardiovascular patients. We thus performed a comprehensive analysis of the short- and long-term outcomes after heart valve surgery with high risk patients.

## Materials and methods

### Study population

High-risk cardiovascular patients scheduled for valve surgery with left ventricular dysfunction (left ventricular ejection fractions < 40%) were screened retrospectively from May 2015 to December 2016. The study was approved by the ethic committee of Cangzhou Central Hospital and all patients signed an informed consent form. Patients requiring emergency surgery and undergoing atrial fibrillation, irregular heart rhythm, unstable hemodynamic state, impaired pulmonary, and renal function were excluded from the study.

Demographic data and risk factors were acquired by directly interviewing with patient before the CCTA evaluations. Low-density lipoprotein cholesterol (LDL-C) higher than 140 mg/dl and high-density lipoprotein (HDL-C) lower than 40 mg/dl was defined as dyslipidemia. Diabetes was defined as hemoglobin A1c (HbA1c) higher than 6.5%. Hypertension was defined as self-reported hypertension history and/or administration of antihypertensive medicines or blood pressure higher than 140/90 mm Hg. Smoking status was classified into never, past, or current smoker (at least 6 months, at least one cigarette per day). Body mass index (BMI) was computed as body weight/height^2^. Heart failure (HF) is generally classified according to the New York Heart Association (NYHA) classification system (I = no limitation of physical activity; II = slight limitation of physical activity; III = marked limitation of physical activity; IV = inability to carry out any physical activity without discomfort) [12].

### Coronary computed tomography angiography

In order to find the link between complications and death after CAD operation, CCTA was utilized for long-term (up to 40 weeks) and time course (10, 20, and 40 weeks) follow-up study. A modified 16-segment American Heart Association coronary tree model was used to detect plaques. The number, presence, characteristics of plaque, and severity of stenosis were assessed by level III equivalent investigators following the Society of Cardiovascular CT (SCCT) guideline. The presence of any plaque, their extent, severity, and type were further defined. The severity of CAD was categorized according to the severity of stenosis, as none (no luminal stenosis), nonobstructive (less than 50% luminal stenosis), and obstructive stenosis (more than 50% luminal stenosis), and then obstructive stenosis was further classified as 1-, 2-, and 3-vessel disease (VD) [13]. The segment involvement score (SIS) was calculated as the total number of coronary artery segments exhibiting plaque which can be used to define the extent of CAD [14], which was categorized into three subgroups with SIS of 0, 1–5, and over 5. Plaque characteristics were categorized as noncalcified plaques (NCAP), calcified plaques (CAP), and mixed calcified plaques (MCAP) [15].

### Complication

Patients were considered to have immediate postsurgery complications if one or more of the following occurred [16–18]: (1) organ-related complications (cardiovascular, respiratory, neurological, and renal dysfunction); (2) complications associated with surgery (including bleeding, thromboembolism, hemolysis, and wound site infection); (3) prolonged intensive care unit admission over 4 days; or (4) death. Patients were deemed to have complications at 10, 20, and 40 weeks postsurgery using the same criteria as described above.

### Statistical analysis

All statistical analyses were performed using SPSS 20.0 statistics package (SPSS, Inc., Chicago, IL). Statistical comparison of parametric data was performed with a two-tailed unpaired Students *t*-test. *P*<0.05 was considered to be statistically significant.

## Results

### Baseline characteristics

Baseline characteristics of 46 recruited patients (average age of 73 years) with left ventricular dysfunction (left ventricular ejection fractions < 40%) was shown in Table 1. Most patients were in NYHA classification III (35/46) and IV (9/46) and three patients (6%) undergone precoronary artery bypass graft surgeries. Serious obstructive CAD was also observed (2-VD, 12/46; 3-VD, 34/46).

**Table 1:** Characteristics of study patients at baseline

	Total
	*n*=46
Age (years)	73 ± 8.26
Male gender	25 (54.35%)
Family history of CAD	15 (32.61%)
Current smoke	18 (39.13%)
Diabetes	27 (58.69%)
Dyslipidemia	21 (45.65%)
Hypertension	29 (63%)
Hypercholesterolemia	36 (78.26%)
BMI (kg/m^2^)	27.34 ± 4.13
Chronic lung disease	7 (15.22%)
Cerebrovascular disease	5 (10.87%)
NYHA Classification	
I	1 (2.2%)
II	1 (2.2%)
III	35 (76%)
IV	9 (19.6%)
No. of lesions	3.2 ± 0.9
No. of grafts	3 ± 2
Echo EF	39 ± 3
CCT parameters	
SIS	12.8 ± 1.2
NCAP	2 (4.3%)
MCAP	8 (17.39%)
CAP	36 (78.31%)
1-VD	0 (0)
2-VD	12 (26%)
3-VD	34 (74%)

Abbreviations: 1-VD, no luminal stenosis; 2-VD, less than 50% luminal stenosis; 3-VD, more than 50% luminal stenosis; BMI, body mass index; CAP, calcified plaques; CCT, coronary computed tomography; MCAP, mixed calcified plaques; NCAP, noncalcified plaques; SIS, segment involvement score.

### Immediate outcomes

Patients (*n*=29) with immediate complications postsurgery were compared to those well-matched subjects (*n*=17) who were complication free (Table 2). Immediate complications group showed high NYHA classification score when compared with no complications group (13.79% vs 5.88% in IV, *P*=0.002; 79.32% vs 76.48% in III, *P*=0.056; 3.40% vs 11.76% in II, *P*=0.02; 3.40% vs 5.88% in I, *P*=0.07). While, there was no significant difference between the two groups in Echo EF, No. of Lesions, and No. of Grafts as shown in Table 2.

**Table 2:** Characteristics of patients with immediate complication and non-complication

	Comp	Non-comp
	*n*=29	*n*=17
Age (years)	75 ± 4.15	70 ± 5.87
Male gender	14 (48.27%)	11 (64.7%)
Family history of CAD	10 (34.48%)	5 (29.41%)
Current smoke	12 (41.38%)	6 (35.29%)
Diabetes	18 (62.07%)	9 (52.94%)
Dyslipidemia	13 (44.82%)	8 (47.06%)
Hypertension	11 (37.93%)	7 (41.18%)
Hypercholesterolemia	23 (79.31%)	13 (76.47%)
BMI (kg/m^2^)	26.8 ± 4.25	27.3 ± 3.67
Chronic lung disease	5 (17.24%)	2 (11.76%)
Cerebrovascular disease	3 (10.34%)	2 (11.76%)
NYHA classification		
I	1 (3.4%)	1 (5.88%)
II	1 (3.4%)	2 (11.76%)
III	23 (79.32%)	13 (76.48%)
IV	4 (13.79%)	1 (5.88%)
No. of lesions	3.4 ± 0.8	3 ± 1
No. of grafts	3.2 ± 0.9	3.1 ± 0.9
Echo EF	36 ± 3	35 ± 4

Abbreviations: BMI, body mass index; comp, patients with immediate complication; non-comp, patients with non-complication.

### Short- and long-term outcomes

Characteristics of patients 10, 20, and 40 weeks after heart valve surgery were shown in Table 3. At this time, Echo EF, No. of Lesions, and No. of Grafts showed no different among different groups. The higher NYHA Classification (III plus IV) was significantly reduced after 10 (*n*=37), 20 (*n*=32), and 40 weeks (*n*=28) after heart valve surgery, and the total proportion was 81.1%, 56.3%, and 32.14%, respectively.

**Table 3:** Characteristics of patients 10, 20, and 40 weeks after heart valve surgery at baseline

	10 weeks	20 weeks	40 weeks
	*n*=37	*n*=32	*n*=28
Age (years)	73 ± 7.23	72 ± 7.38	73 ± 4.76
Male gender	20 (54.05%)	17 (53.13%)	14 (50%)
Family history of CAD	11 (29.73%)	9 (28.12%)	7 (25%)
Current smoke	13 (35.14%)	10 (31.25%)	6 (21.43%)
Diabetes	20 (54.05%)	18 (56.25%)	15 (53.57%)
Dyslipidemia	17 (45.95%)	14 (43.75%)	12 (42.86%)
Hypertension	13 (35.13%)	10 (31.25%)	9 (32.14%)
Hypercholesterolemia	27 (72.97%)	22 (68.75%)	19 (67.85%)
BMI (kg/m^2^)	25.28 ± 6.44	26.87 ± 5.45	27.36 ± 7.12
Chronic lung disease	6 (16.22%)	4 (12.5%)	3 (10.71%)
Cerebrovascular disease	5 (13.51%)	4 (12.5%)	4 (14.28%)
NYHA classification			
I	2 (5.4%)	4 (12.5%)	5 (17.86%)
II	5 (13.5%)	10 (31.2%)	14 (50%)
III	27 (72,9%)	17 (53.2%)	8 (28.57%)
IV	3 (8.2%)	1 (3.1%)	1 (3.57%)
No. of lesions	3.1 ± 0.6	3.2 ± 0.8	3.4 ± 0.3
No. of grafts	3.2 ± 0.7	3.1 ± 0.8	3 ± 1
Echo EF	36 ± 2	33 ± 4	32 ± 2

Abbreviation: BMI, body mass index.

**Figure 1:**
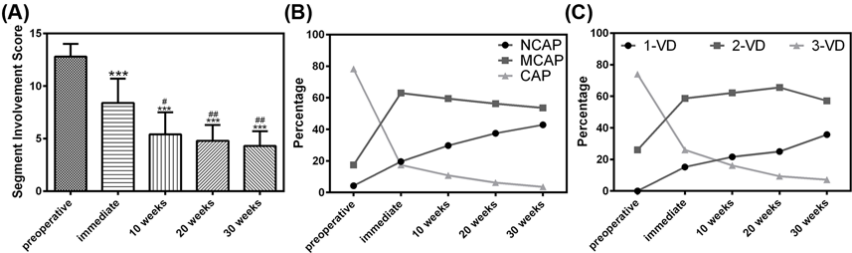
Coronary computed tomography parameters examined in the particpants. Coronary computed tomography parameters in patients preoperative, immediate, 10, 20, and 40 weeks after heart valve surgery, including segment involvement score (**A**), coronary artery plaque type (**B**) and severity of CAD (**C**); ***P<0.001 compared with preoperative segment involvement score; #*P*<0.05 and ##*P*<0.01 compared with immediately after surgery.

**Figure 2:**
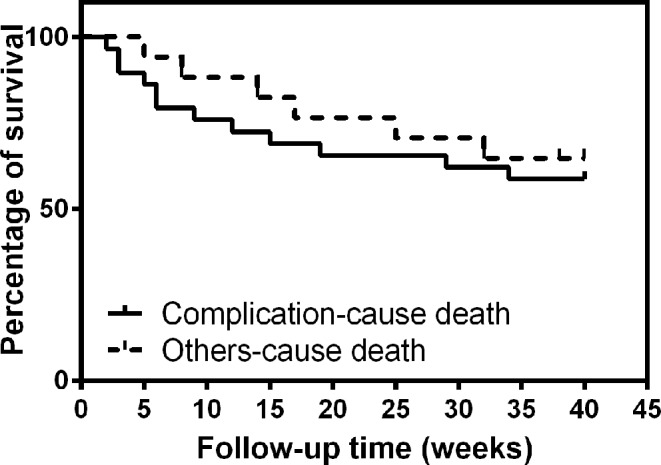
Survival examination in the participants. Free survival curves of complication-cause death and other-cause death.

It was noted that among the CCTA parameters we detected, SIS, MCAP, CAP, and 3-VD showed decreased trend from immediate time after surgery to 10, 20, and 40 weeks following the surgery (Table 4 and Figure 1), NCAP, 1-VD showed increased trend, and SIS was the only indicator did reach statistical significance when 10, 20, and 40 weeks after surgery compared with immediate measure (Figure 1). The cumulative mortality rate at 10, 20, and 40 weeks were 19.56%, 30.43%, and 39.13%, respectively (Table 3 and Figure 2). Free survival curves of complication-cause death and other-cause death were shown in Figure 2. It can be seen that the early death is mainly due to complications, and with the time-lapse of surgery, the impact of complications on death is gradually eliminated.

**Table 4:** Coronary computed tomography parameters in patients immediately, 10, 20, and 40 weeks after heart valve surgery

	Immediate	10 weeks	20 weeks	40 weeks
	*n*=46	*n*=37	*n*=32	*n*=28
SIS	8.4 ± 2.3	5.4 ± 2.1	4.8 ± 1.5	4.3 ± 1.4
NCAP	9 (19.56%)	11 (29.73%)	12 (37.5%)	12 (42.85%)
MCAP	29 (63%)	22 (59.46%)	18 (56.25%)	15 (53.57%)
CAP	8 (17.44%)	4 (10.81%)	2 (6.25%)	1 (3.58%)
1-VD	7 (15.22%)	8 (21.62%)	8 (25%)	10 (35.71%)
2-VD	27 (58.69%)	23 (62.16%)	21 (65.63%)	16 (57.14%)
3-VD	12 (26.09%)	6 (16.22%)	3 (9.37%)	2 (7.15%)

Abbreviations: 1-VD, no luminal stenosis; 2-VD, less than 50% luminal stenosis; 3-VD, more than 50% luminal stenosis; CAP, calcified plaques; MCAP, mixed calcified plaques; NCAP, noncalcified plaques; SIS, segment involvement score.

## Discussion

For all we know, it is the first time that CCTA was specifically utilized to track postoperative patients after CAD and to assess short- and long-term outcomes at multiple time points (10, 20, and 40 weeks). And the link between complications and postoperative death was also traced. SIS, one of CCTA parameters after 10, 20, and 40 weeks after heart valve surgery, showed statistical significance when compared with baseline, which indicated that SIS might be associated with the short- and long-term outcomes after heart valve surgery in high risk cardiovascular patients with left ventricular dysfunction, concomitant coronary artery disease, and or preoperative symptoms of advanced congestive heart failure and SIS might be a predictor of outcomes.

Consider the compliance of the patient, invasive detecting methods cannot be used as a routine method during the follow-up period due to potential serious complications. Recent developments in CCTA have made imaging of the coronary arteries possible, and when comparison with traditional invasive coronary angiography, the sensitivity of CCTA ranging from 83% to 99%, specificity between 93% and 98%, and negative predictive value from 95% to 100% have been reported for the detection of coronary artery stenoses, all of which suggest that CCTA may be a useful follow-up technique [19,20].

Most studies are performed to stratify CAD using CCTA to predict the outcome of heart valve surgery. Some studies showed that transcatheter aortic valve replacement patients stratified according to the extent of CAD demonstrated similar survival rates, and other studies showed that concomitant CAD in patients undergoing valve replacement increased mortality [21–24]. Such contradiction can be attributed to the heterogeneity of data on the anatomic and physiological burden of CAD, which make it difficult to interpret the direct impact of CAD on the short- and long-term outcomes of heart valve surgery. In our research, the extent of CAD was defined by SIS, the severity of CAD was subcategorized as 1-, 2-, and 3-VD, and plaque characteristics were categorized as NCAP, MCAP, and CAP. Such attempt to clearly define the CAD characteristics might be a real asset to associated research.

In our study, CCTA was applied to detect the short- and long-term outcomes after heart valve surgery with high risk patients, and such noninvasive detection can be very significant to prognosis of heart valve surgery. Among the CCTA parameters detected after 10, 20, and 40 weeks after heart valve surgery, only SIS did reach statistical significance when compared with baseline levels, all of which was also supported by other research that SIS on CCTA, an indicator of the extent of CAD, was a strong, independent predictor of cardiovascular events [25]. However, the study we performed was limited by small sample sizes in single center, which might introduce bias that obfuscates the actual diagnostic or predictable performance of CCTA, further multicenters investigations on a large scale of participants are needed to confirm the present results. While, in order to get more accurate diagnosis potential, more advanced imaging postprocessing methods are needed to give not only morphological information but also functional abnormalities.

In summary, we demonstrate the possibility of using CCTA to detect short- and long-term outcomes after heart valve surgery with high risk cardiovascular patients in Chinese for the first time. It can be seen that the impact of complications on death is gradually eliminated with the time-lapse of surgery and SIS level is associated with the short- and long-term outcomes.

## Conclusions

Although need further confirmed by multicenters investigations, our analysis demonstrates that CCTA might be a useful alternative tool to detect short- and long-term outcomes after heart valve surgery in high risk cardiovascular patients, to whom, noninvasive detection is more appropriate.

## Abbreviations

CAD: coronary artery disease
CCTA: coronary computed tomography angiography
NYHA: New York Heart Association
SIS: segment involvement score
